# Exploiting Cell-To-Cell Variability To Detect Cellular Perturbations

**DOI:** 10.1371/journal.pone.0090540

**Published:** 2014-03-04

**Authors:** Gautam Dey, Gagan D. Gupta, Balaji Ramalingam, Mugdha Sathe, Satyajit Mayor, Mukund Thattai

**Affiliations:** 1 Stanford University, Palo Alto, California, United States of America; 2 Samuel Lunenfeld Research Institute, Toronto, Canada; 3 Centre for Cellular and Molecular Platforms (C-CAMP), Bangalore, India; 4 National Centre for Biological Sciences, Tata Institute of Fundamental Research, UAS/GKVK Campus, Bangalore, India; University of Texas School of Public Health, United States of America

## Abstract

Any single-cell-resolved measurement generates a population distribution of phenotypes, characterized by a mean, a variance, and a shape. Here we show that changes in the shape of a phenotypic distribution can signal perturbations to cellular processes, providing a way to screen for underlying molecular machinery. We analyzed images of a Drosophila S2R+ cell line perturbed by RNA interference, and tracked 27 single-cell features which report on endocytic activity, and cell and nuclear morphology. In replicate measurements feature distributions had erratic means and variances, but reproducible shapes; RNAi down-regulation reliably induced shape deviations in at least one feature for 1072 out of 7131 genes surveyed, as revealed by a Kolmogorov-Smirnov-like statistic. We were able to use these shape deviations to identify a spectrum of genes that influenced cell morphology, nuclear morphology, and multiple pathways of endocytosis. By preserving single-cell data, our method was even able to detect effects invisible to a population-averaged analysis. These results demonstrate that cell-to-cell variability contains accessible and useful biological information, which can be exploited in existing cell-based assays.

## Introduction

Advances in labeling and imaging have made it possible to collect quantitative information on a variety of cellular processes at single-cell resolution, and to generate high-quality population distribution data. Cell-to-cell variability is evident in any such measurement [1], [2]. This variability plays an essential role in processes ranging from bet-hedging in unicellular organisms, to cell differentiation, ageing, and infection in metazoa [3]–[7]. Cell-to-cell variability can be generated by intrinsic stochastic mechanisms and shaped by regulatory molecular circuitry: transcriptional regulation impacts protein expression distributions in bacteria and yeast [8]–[10]; the molecular machinery controlling cell shape generates morphological variability in metazoan cells [11], [12]; DNA-damage checkpoints produce noisy oscillations in cancer cell lines [13]. A corollary of these results is that the entire population distribution of a phenotype can be used to study the underlying biology. Here we present an explicit demonstration of this idea: rather than investigating how specific molecular mechanisms generate variability, we reverse the process and use variability itself as a general probe of those mechanisms. We first show that genetic perturbations reliably cause changes to the population distributions of a variety of phenotypes related to cell morphology and activity. We then demonstrate how such changes in phenotypic distributions can be exploited, in conjunction with a screening approach such as genome-wide RNA interference (RNAi), to probe cellular processes with unprecedented sensitivity. An application of this method, to study the molecular basis of multiple endocytic pathways in metazoan cells, is reported in a companion paper [14].

Image-based RNAi screens have previously been used to understand the molecular basis of diverse cellular processes, including: pathogen entry mechanisms; intracellular traffic; cell motility, growth, and differentiation; and cell death and aging [15]–[18]. Though these screens often collect data at single-cell resolution [19]–[24], they invariably focus on population-averaged values to select hits, and rely on heuristic normalization techniques to compensate for labeling and imaging artifacts which cause these values to be erratic [25], [26]. Here we use a radically different strategy, which paradoxically *relies* on the occurrence of cell-to-cell variability: we focus purely on the shapes of population distributions which, as we show, are robust against measurement artifacts. The shapes of these distributions change under RNAi down-regulation; we quantify these changes, and thus identify genes which influence various cellular phenotypes. This shape-based strategy complements existing methods for analyzing image-based screens. It can be directly applied to any perturbation experiment which generates single-cell data, for a variety of phenotypes, and is able to identify subtle hits which are missed using standard approaches.

## Results

### Single-cell-resolved features from an image-based RNAi screen

**Figure 1:**
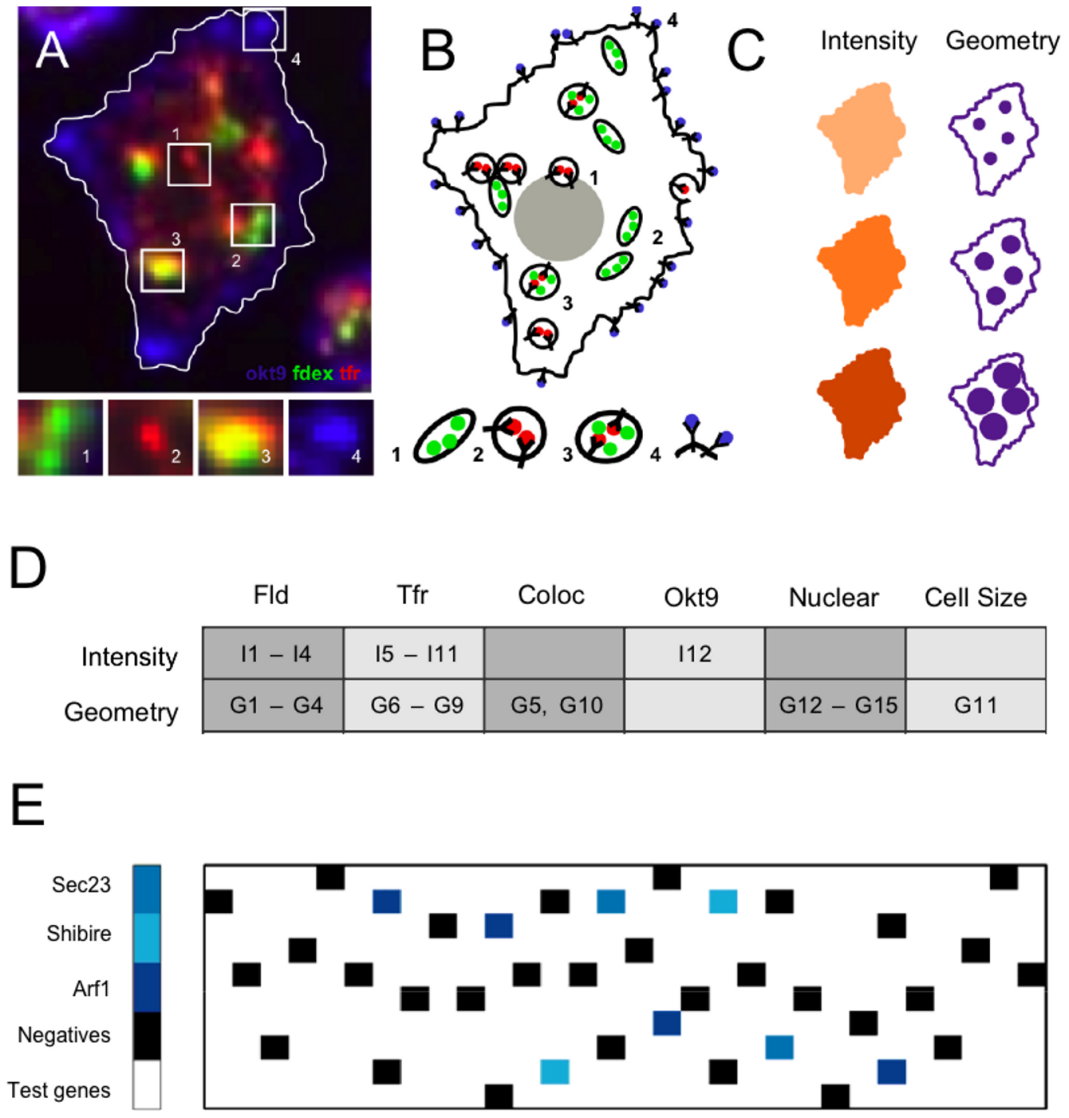
An image-based RNAi screen for endocytic and cell morphological features. (**A**) A single *Drosophila* S2R+ cell, fixed and imaged at 20X and 0.75 NA; the scale bar is 3 µm. FITC-Dextran (green) labels the GEEC pathway responsible for fluid-phase uptake; Alexa568-Transferrin (red) labels the clathrin-dependent receptor-mediated endocytic pathway; the Alexa647-Okt9 antibody (blue) labels steady-state cell surface levels of the Transferrin receptor; the nucleus (not shown) was imaged with a Hoechst stain. Region 1: pure GEEC endosome; Region 2: pure Transferrin endosome; Region 3: colocalization signature, marking a heterotypic fusion product between the two types of endosomes; Region 4: surface cluster of Okt9. (**B**) Schematic representation of the cell from Figure 1A. (**C**) Schematic representation of intensity features (orange), which track the cell-averaged intensity of the various fluorescent labels; and geometric features (purple), which track the sizes and shapes of the cell, of the nucleus, and of endosomes. (**D**) 27 single-cell features. The two rows correspond to intensity and geometric features; each column relates to individual endocytic pathways or cell-morphological features. See Table S1 in File S1 for detailed feature descriptions. (**E**) The screen was carried out on glass slides printed with 300 wells in a 10×30 format, each well containing dsRNA targeted against different genes. Colors represent negative (black) or positive (blue) control wells, while white represents test wells. Details of the image analysis and the experimental conditions are provided in the companion paper [14].

We applied these ideas in the context of an image-based RNAi screen, with the goal of identifying molecular machinery involved in multiple pathways of metazoan endocytosis. The results of this endocytic screen are presented in the companion paper [14]; here we focus on the use of cell-to-cell variability as a general cell-biological probe. We simultaneously tracked two endocytic pathways [27] in Drosophila S2R+ cells using a pulse-labeling assay [28]: the clathrin- and dynamin-independent CLIC/GEEC endocytic pathway [29] responsible for fluid-phase uptake (probed using FITC-conjugated dextran; green, Fig. 1A,B); and the canonical clathrin-dependent pathway [30] responsible for receptor-mediated endocytosis (probed by using Alexa568-conjugated Transferrin, which is taken up by an ectopically expressed human Transferrin receptor [28]; red, Fig. 1A,B). We used fluorescence microscopy and automated image analysis to extract 27 features for each cell (see Table S1 in File S1, and companion paper [14] for further details): 12 intensity-dependent features describing total uptake levels of the two endocytic probes, and surface levels of the Transferrin receptor as labeled by the monoclonal antibody Okt9 [28] (orange cartoon, and I1-I12; Fig. 1C,D); and 15 geometric features quantifying the shape, size and number of endocytic compartments, as well as nuclear and cell morphology (purple cartoon, and G1-G15; Fig. 1C,D). The screen was performed on custom-designed glass slide arrays of 300 wells printed with double-stranded RNA (dsRNA) (Fig. 1E): 30 wells were negative controls (15 with no dsRNA and 15 with dsRNA targeting the gene for zeocin resistance, which is absent in the Drosophila genome; black in Fig. 1D); 8 wells were positive controls (dsRNA targeting Shibire [31] for the receptor-mediated pathway, and dsRNA targeting Sec23 and Arf1 for the fluid-phase GEEC pathway [28]; shades of blue in Fig. 1D); the remaining wells contained dsRNA targeting individual genes to be screened. Each slide was assayed in triplicate, with scrambled dsRNA patterns (Fig. S1A in File S1). Cells were grown for four days in the presence of dsRNA, then fixed and imaged. We tested a total of 7216 dsRNAs targeting 7131 unique genes.

### Cell-to-cell and well-to-well variability in feature distributions

**Figure 2:**
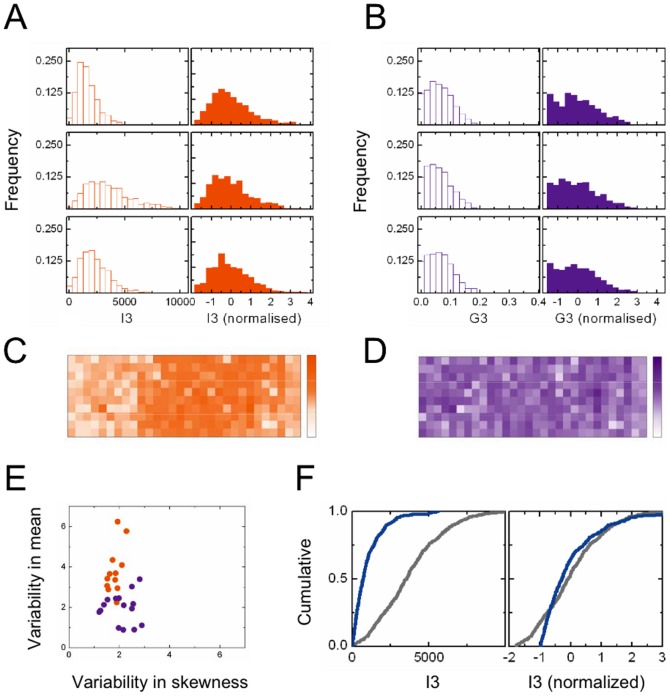
Well-to-well and cell-to-cell variability. (**A,B**) Population distributions (histograms) of features I3 (A) and G3 (B), for three negative control wells from a single slide. Hollow bars show raw distributions; solid bars show the data when distributions are normalized to have zero mean and unit variance. (**C,D**) Heat maps of population-averaged mean values for features I3 (C) and G3 (D). The positional effects in Figure 2C likely arise from labeling and imaging artifacts. (**E**) ANOVA F-statistic for inter-row variance versus within-row variance of distribution means (x-axis) or skewnesses (y-axis), for all 27 features. Each point shows the median F-statistic over 84 slides; intensity features are colored orange, geometric features are colored purple. Data for each slide are shown in Figure S1B,C in File S1. (**F**) Cumulative distributions of feature I3, from a negative control well (grey) and a positive control well (Arf1; blue). The left panel shows raw data; the right panel shows that cumulative distributions are still distinguishable after normalization.

We measured phenotypic distributions of the 27 single-cell features for each well (hollow histograms, Fig. 2A,B), with population sizes ranging from 200 to 500 cells. All the distributions we measured showed significant cell-to-cell variability; but we also saw a great deal of well-to-well variability between replicate measurements. Even among negative controls, feature distributions were erratic, though geometric features were typically more robust than intensity features (compare hollow purple histograms in Fig. 2B to hollow orange histograms in Fig. 2A) with the latter showing significant slide-positional artifacts (orange heat map, Fig. 2C). To further quantify this effect we carried out 1-way ANOVA [32] for two scale-related measures (mean and variance) and two dimensionless shape-related measures (skewness and kurtosis) of each distribution, using the ANOVA F-statistic to compare the variability of these measures between and within the rows of each slide. This analysis confirmed (Fig. 2E) that the mean and variance of intensity features (but not of geometric features) were susceptible to row-dependent artifacts, but shape-related measures for all features less so. The same trends were observed for variability between columns of a slide and between entire slides, and between negative controls on different slides, across all intensity and geometry features (Fig. S1B,C in File S1).

Based on these results, we surmised that the well-to-well differences in the mean and variance of intensity features had their origin in background (additive) and scale (multiplicative) artifacts (from dye loading or illumination, for example) which don’t influence geometric measurements. These artifacts should neutralized by the standard procedure of subtracting the mean and dividing by the standard-deviation, leaving a distribution that is characterized by its shape alone. Indeed, phenotypic distributions from replicate measurements converged to nearly identical shapes once normalized in this way, for both intensity and geometry features (filled histograms, Fig. 2A,B). However, if the feature axis was re-scaled non-linearly before normalization (such as by a logarithmic or power-law transformation), geometric distributions typically converged while intensity distributions did not. This provided further evidence that normalization was working to counter affine (additive and multiplicative) artifacts.

### Feature distributions change shape under perturbations

**Figure 3:**
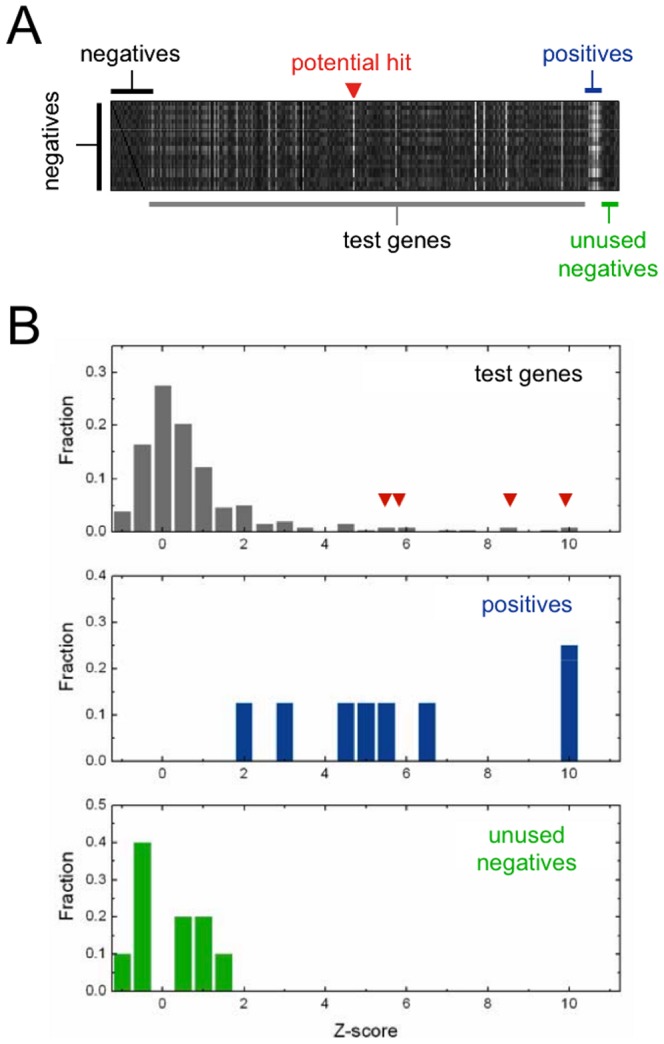
Calculating Z-scores to quantify shape changes. (**A**) A heatmap of all pair-wise KS-statistics for all 300 wells on a representative slide (columns) against negative controls (rows), for feature I3. The genes have been sorted horizontally, starting with 20 selected negatives (black), test genes (grey), positives (blue), and 10 unused negatives (green). Lighter boxes indicate higher shape deviations; the negatives tested against themselves show the lowest scores (dark sections on the left and right), while the positives show the highest scores (white vertical stripe). A potential hit (red triangle) shows up as a white vertical line. (**B**) Histogram of Z-scores for the slide depicted in (A), with test genes, positives and unused negatives plotted separately. Potential hits are marked by red triangles. The final selection of a hit depends on how a gene performs in triplicate.

**Figure 4:**
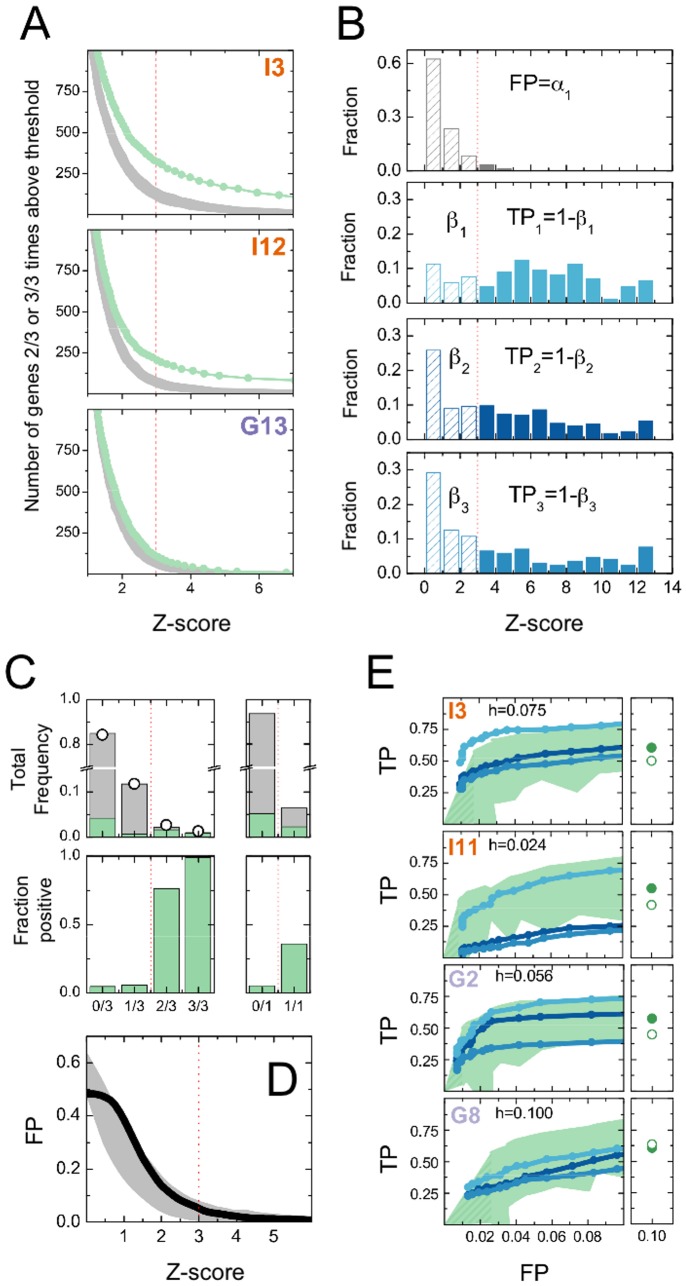
Statistical performance of shape-based scoring. (**A**) The number of genes that occur two or more times above each Z-score threshold, for three representative features. The green curve shows the number of genes selected from the screen; the grey band represents the upper and lower limits of number of genes selected from 1000 randomly permuted datasets. We used a Z-score cutoff of 3 (red line) to select hits. (**B**) For feature I3, distribution of Z-scores for negative control wells (top panel), and positive control wells (second panel: Shibire; third panel: Arf1; fourth panel: Sec23). At a given Z-score threshold (red line), the false-positive rate (FP  =  *α*) is the fraction of negatives above threshold (solid grey bars), while true-positive rate (TP  =  1-*β*) is the fraction of positives above threshold (hollow blue bars). (**C**) For feature I3, the upper left panel shows the fraction of genes occurring zero, one, two, or three times above a Z-score threshold of 3; circles show actual data, bars show the inferred composition of each bin, in terms of positives (green) and negatives (grey). The lower-left panel shows the fraction of positives in each bin; genes occurring two or more times above threshold are strongly enriched in positives. The two right panels show the performance when hits are selected from a single measurement rather than using triplicates. (**D**) The grey band shows the range of inferred FP rates for 25 features (excepting features G11 and G15 for which the inference procedure fails to converge); the black line shows the mean of the measured FP rates for the same features. (**E**) Inferred TP rates. Green bands show the range of inferred true positive rates (1–*β_0_* ± *σ*; see Methods**:** Assessing statistical power from triplicate data) as a function of inferred false positive rates (*α*); blue lines show the observed TP and FP rates among positive control genes (light: Shibire; medium: Sec23; dark: Arf1). The box to the right of each graph shows the inferred average TP rate (1–*β_0_*) at FP  =  0.1; the solid dot shows the performance using normalized distributions, the hollow dot shows the performance using un-normalized distributions.

Our key observation was that positive and negative control wells could be distinguished even after normalizing out mean and variance (Fig. 2F); the resulting distributions are characterized entirely by their shapes. We used a Kolmogorov-Smirnov-like (KS) statistic [32], [33] to assign a Z-score to each gene on a slide (Fig. 3; Methods: A Z-score to quantify shape changes of phenotypic distributions). This Z-score quantifies distribution shape changes between test and negative control wells; the higher the score, the greater the shape deviation. Since each gene was tested in triplicate, we calculated three Z-scores for every gene, and pooled these data over the entire screen. We classified a gene as a hit if it occurred two or more times above a given Z-score threshold (Fig. 3B). Figure 4A shows the number of hits selected from the screen (green curve) compared to the number of hits selected from randomly permuted genes (grey band) as the Z-score threshold is varied. The deviation of the green curve from the grey band reveals the presence of reproducible hits in the dataset. The maximal deviation occurs near a Z-score threshold of 3 across all features (Fig. S2A in File S1). Using this threshold we identified 1072 unique genes as hits for one or more features [14].

### Shape-based scoring reveals a spectrum of weak-to-strong genetic contributions

The response to perturbations over triplicate measurements revealed an unexpectedly complex connection between genes and phenotypes. For each feature, at a given Z-score threshold we can classify genes into four bins: those occurring either zero, one, two, or three times above threshold. Each of these bins will contain a combination of true hits and negatives. We can infer false-positive rates (FP: the fraction of above-threshold negatives; Fig. 4B, top panel) and true-positive rates (TP: the fraction of above-threshold hits; Fig 4B, bottom three panels) by fitting the observed gene number in each bin to a statistical model (Fig. 4C; Methods: Assessing statistical power from triplicate data). The inferred FP rates matched well to the FP rates measured for negative controls (Fig. 4D). However, we were not able to infer a single TP rate consistent with the data. The behavior of the positive controls highlights the problem: different genes appear to have different, characteristic TP rates (Fig. 4B).

Extending this idea, we postulated that hits over the entire screen had a *distribution* of TP rates. Under this assumption, it was possible to fit all the observed data, and therefore to infer FP rates (Fig. 4E, x-axes) and the range of TP rates (Fig. 4E, green band along y-axes) as the Z-score threshold was varied. For most features, a Z-score threshold of 3 corresponds to FP < 0.1 and TP ∼ 0.5 for single measurements; the FP rate is lower and the TP rate higher if we use triplicate data with a 2/3 rule (Fig. 4C). In support of our calculation, measured TP rates from different positive controls fell within the inferred band of TP rates (Fig. 4E, solid lines; Fig. S3 in File S1). The broad distribution of TP rates is a property of the underlying biology, related to the varying degrees of influence different genes can have on the phenotype of interest.

### Shape-based scoring outperforms other scoring methods

**Figure 5:**
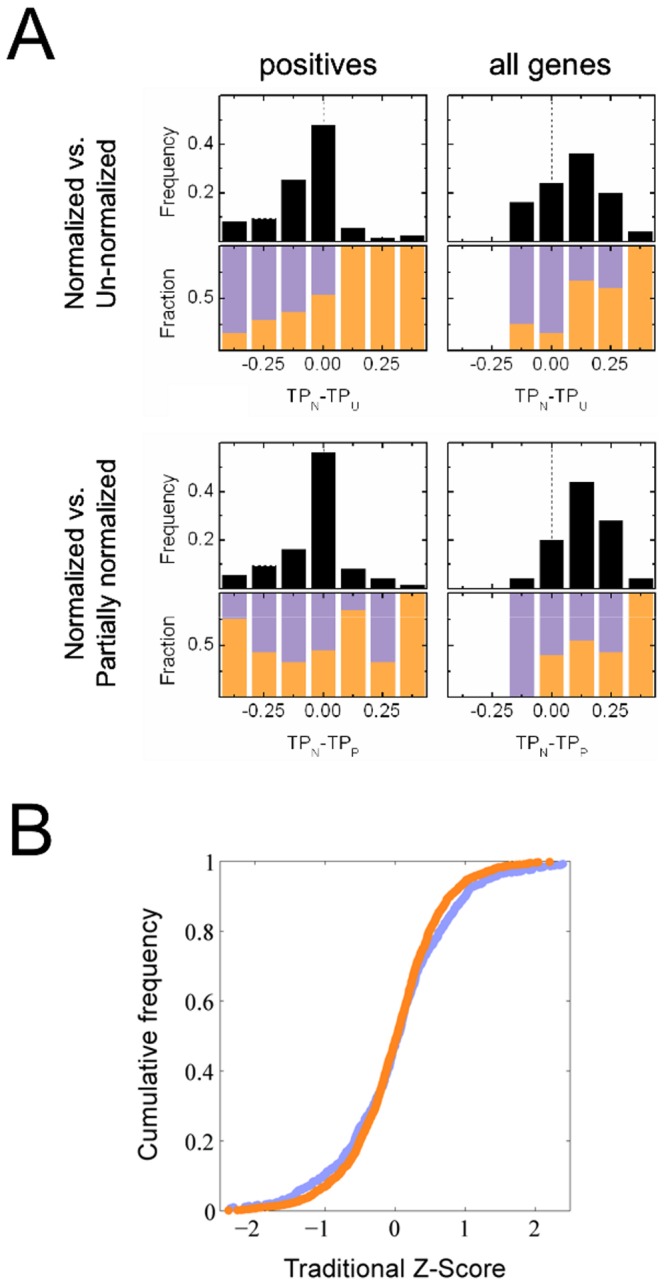
Comparing shape-based scoring to other scoring methods. (**A**) The difference in the true-positive rate TP_N_–TP_U_ represents the increase in performance derived from normalization. Upper panels: black histograms show the distribution of TP_N_–TP_U_ values for 25 features (excepting G11 and G15); colored bars represent the fraction of intensity (orange) and geometric (purple) features in each bin. The left panel shows the observed performance for positive controls; the right panel shows the inferred performance over all genes. Lower panels: same as upper, but now the normalization strategy (TP_N_) is compared to partial normalization (TP­_P_), when bins are normalized to have the average mean and variance of their eight nearest neighbors. (**B**) The ‘traditional’ Z-score is defined based only on the mean values of feature distributions. The figure shows the cumulative distributions of traditional Z-score values for genes that have been validated as hits for intensity features (orange) or geometric features (purple) in an independent experimental assay. Less than 10% of these Z-scores have an absolute value greater than unity.

There are many possible variations of the KS-based Z-score, differing on how the phenotypic distributions are initially modified. We compared three options: (U) Un-normalized or raw distributions; (P) Partially normalized distributions, transformed to have the average mean and variance of nearest neighbors; (N) Normalized distributions, transformed to have zero mean and unit variance. We evaluated the observed true positive rate (assuming all positive controls are hits for all features), and the inferred mean true positive rate (from triplicate data) at a false positive rate FP  =  0.1, and calculated the improvements in performance between different methods: TP_N_ – TP_U_ and TP_N_ – TP_P_. The values for 25 features (excepting G11 and G15 for which triplicate inference failed) are binned into histograms in Figure 5A; the bar graphs indicate the fraction of intensity (orange) or geometric (purple) features in each bin; left panels show observed improvements for positive controls, right panels show inferred improvements for all genes. We find that for intensity features, which are plagued by measurement artifacts, normalization actually increases the power of the screen; for geometric features, which are less susceptible to artifacts, our method performs at least as well using normalized distributions as using raw distributions. Specifically: at FP  =  0.1, the inferred average TP rate with normalization (that is, using shape alone; Fig. 4E, solid green dots) exceeds that without normalization (Fig. 4E, hollow green dots) for all intensity features and some geometric features (Fig. 5, top panels). We did however find that normalization typically does not improve or harm performance when applied to positive controls (Fig. 4, left panels) which are associated with strong perturbations.

**Figure 6:**
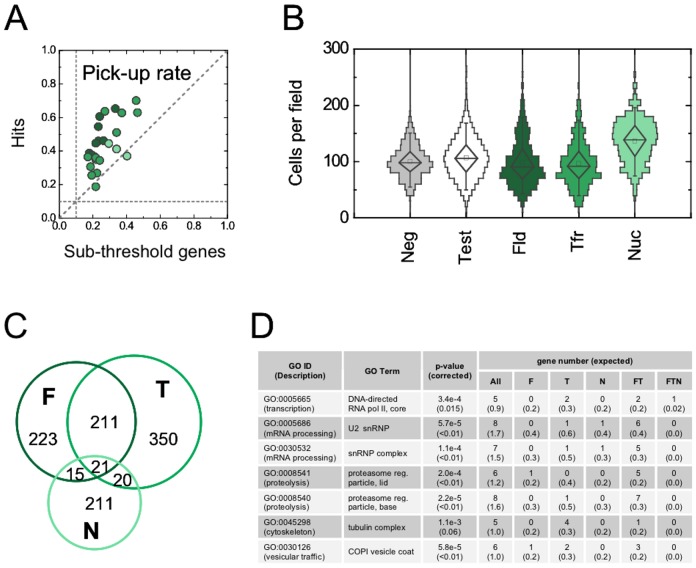
Results and biological significance. (**A**) We validated hits using an independent experimental assay based on the population-averaged mean value of each phenotype [14]. We carried out this measurement both for hits as well as for a number of non-hits (genes with below-threshold Z-scores). The y-axis shows the fraction of original hits validated (FP  =  0.1); the x-axis shows the fraction of sub-threshold genes validated. Each dot gives the result for a single feature type; hits for features G11 and G15 were not included in the secondary measurement. Horizontal and vertical dotted lines show the FP rate. The distinction between hits and sub-threshold genes was based on shape-based scoring alone, but the former are detected at a much higher rate than the latter using the population-average-based assay. This demonstrates a strong correlation between the ability of a gene to influence the mean value of a phenotype and the shape of a phenotypic population distribution. (**B**) Relationships of hit subsets to cell density. We show the cell number per imaging field as a vertical histogram. The median (horizontal line), mean (box), and percentiles (5%, 25%, 75%, 95%) of the cell number distribution are overlaid. Histograms are separately shown for negative control wells, all test wells, and for fluid uptake, Transferrin uptake, and nuclear morphology hits. (**C**) Area-proportional Venn diagram of hits that influence fluid-phase uptake (F), Transferrin-receptor-mediated uptake (T), or nuclear and cell morphology (N). Of the 26 genes that influence cell size, 21 which do not influence other features have been omitted. Numbers give the sizes of non-overlapping subsets. The total number of hits is 1051 (shown) + 21 (not shown). (**D**) Functional enrichment. We annotated genes according to the Gene Ontology (GO) ‘cellular component’ classification system, using only the most specific term for each gene. We used the one-tailed Fisher’s exact test to determine an enrichment p-value for each teach GO term among the 1072 hits, given its background occurrence among the 7216 RNAi probes. To correct p-values for multiple hypotheses, we used 1000 simulated datasets in which GO terms had been randomly permuted. The table shows the seven GO terms with corrected p-values < 0.1, along with the observed and expected number of genes among the seven non-overlapping gene subsets of the Venn diagram.

We next compared the performance of the KS-based Z-score with that of a Z-score where the ‘signal’ is restricted only to the mean values of feature distributions. This ‘traditional’ Z-score is defined as the squared difference between the signal of a given well and the average signal of the wells on a given slide, normalized by the variance of all the signals. As before, we can use triplicate data with a 2/3 rule to select hits; Figure S2B in File S1 shows the number of hits selected using the traditional Z-score (green curve) compared to the number of hits selected from randomly permuted genes (grey band). Unlike the KS-based Z-score (Fig. S2A in File S1), at no threshold does the number of hits selected using the traditional Z-score rise above that expected by chance. For further validation, we screened a subset of predicted hits using an independent experimental assay. This validation assay was designed to minimize positional artifacts at the expense of lower throughput, so the mean values of feature distributions could be used to select hits [14]. We found that for both intensity and geometric features, less than 10% of the traditional Z-scores of validated genes exceeded unity in absolute value; most of these hits would have been completely missed by the high-throughput screen. Conversely, there was a strong overlap between genes selected by shape-based scoring with those selected from the mean-value analysis in the validation assay; that is, the hits which had been selected only for their ability to modify the shapes of phenotypic distributions had a strong tendency to perturb the means of those distributions as well (Fig. 6A).

### Biological relevance of shape-based scoring

We applied three independent criteria to test whether our shape-based scoring strategy generates biologically meaningful information. First, we examined whether our hit selection might have been influenced by overall cell health and proliferation, using cell density as a proxy. We found that the cell densities observed in negative control wells broadly overlapped with those in test wells and in wells corresponding to hits. This suggests that the observed changes in the shapes of phenotypic distributions do not arise from gross deficiencies in cell health due to RNA interference. However, there were subtle differences in cell density between hit subsets (Fig. 6B): the number of cells per field among fluid and Transferrin uptake hits (medians 90 and 92, respectively) were only slightly below those among negative control wells (median 98), but nuclear hits showed a striking correlation with high cell number (median 139). This suggests an unexpected connection between nuclear morphology and proliferative capacity, though this must be further explored to rule out confounding factors. Second, we checked whether individual genes influenced multiple types of features in the expected manner. Of the 1072 hits, 470 influenced fluid-phase uptake, 602 influenced Transferrin uptake, 267 influenced nuclear morphology, and 26 influenced cell size (Fig. 6C). Consistent with the expectation that different endocytic pathways share core molecular machinery, there was a high degree of overlap between the fluid and Transferrin uptake gene sets (27%: 211 genes compared to 34 expected by chance). In contrast, there was no significant overlap between endocytic hits and those influencing nuclear morphology (5%: 56 genes compared to 31 expected by chance). Third, we asked whether the lists of hits were enriched for protein complexes, as defined by the Gene Ontology [34] ‘cellular component’ classification system (Fig. 6D). Protein complexes involved in basic cellular processes such as transcription, mRNA processing, and proteolysis, all emerged as strong hits; we also found components of the cytoskeletal and traffic machinery enriched among endocytic hits. These complexes are expected to play a pervasive role in cellular processes; what is surprising is that they can be detected through their influence on the *shapes* of phenotypic distributions alone. By all these criteria our shape-based analysis appears to be well suited to probe the entire spectrum of genes involved in complex cellular processes, covering genes with both subtle and strong effects across a variety of phenotypes.

### A ‘cell state’ model for feature distribution shape changes

We have seen that population distributions change shape when a perturbation is applied; this suggests that different cells in the population might be in distinct states, causing them to respond differently to the perturbation. We can define this hidden ‘cell state’ [35] to be a feature with the following properties: (1) it is not itself affected by a perturbation; (2) it can be used predict the response of some other feature to that perturbation. We refer to such a cell-state feature as a “classifier”, and to the feature being perturbed as the “output”. We searched for potential classifier-output pairs among the 12 intensity features, as well as the cell-size feature (geometric features had narrow or discrete distributions, and were therefore poor candidates for classifiers). Cells were sorted into three classifier percentile bins (A: 5­%–15%, B: 45%–55%, C: 85%-95%) and the mean value of the output was calculated for each bin (*m*_A_, *m*_B_, *m*_C_). These were used to define a correlation score: *y*  =  (*m*_A_ – *m*_B_)/(*m*_B_– *m*_C_). For a given classifier-output candidate pair { *i*, *j* }, we calculated the median value of *y* over all positive control wells, and subtracted from it the median value over all negative control wells, to get the final score Δ*y_ij_*. This 13×13 matrix is shown as a heatmap (Fig. S4A in File S1) separately for the positive controls Arf1, Shi, and Sec23. A feature *i* will be a good candidate for a classifier if the diagonal entry Δ*y_ii_* is close to zero (since it must be unaffected by the perturbation); a corresponding feature *j* is a good candidate for the output if the entry Δ*y_ij_* is high in magnitude, which occurs when the three binned populations respond differentially to RNAi. One candidate pair is shown in the Shibire heatmap (Fig. S4A in File S1): the classifier is cell size (G11) and the output is the intensity feature I9. To explore this classifier-output pair further, we carried out pairwise KS-tests of Shibire-RNAi wells against negative control wells, for both I9 and G11 (Fig. S4B in File S1). It is clear that I9 is strongly affected by the perturbation, but G11 is not. The same result was evident when we pooled data from group of similar wells (Fig. S4B in File S1, red box) and examined the distributions of G11 and I9 without (Fig. S4C in File S1, grey histogram) and with (Fig. S4C in File S1, blue histogram) the RNAi perturbation. We next split the cells into five classifier percentile bins, and examined the mean value of the output in each bin without (Fig. S4C in File S1, grey curve) and with (Fig. S4C in File S1, blue curve) RNAi. We found that the response of the output to RNAi depended strongly on cell size. This implies that the variation in response is at least partly due to the background variation in cell size; however, most of the variation remains unexplained. This is not surprising, since we have only tested a handful of features for explanatory value.

## Discussion

Cell biologists, when limited by the sensitivity of their assays, are sometimes forced to pool data from large numbers of cells. For example, transcriptional, proteomic, or metabolic analyses require large sample sizes in order to enhance signal-to-noise and thus ensure reproducibility. Behind the interpretation of such measurements is the tacit assumption that population averages accurately reflect the state of individual cells. However, as single-cell-resolved data have become more widely available, the “myth of the average cell” has been found to be a poor reflection of reality [1]. Cell-to-cell variability is ubiquitous, and population averages blur much of the complexity of cell-biological phenomena. Several elegant studies have begun to reveal details about the processes which give rise to this variability. Here we have taken a complementary approach, using cell-to-cell variability itself as the cell-biological probe. Rather than relying on traditional population-averaged measures to detect perturbations, this is precisely the information we ignore – we normalize all our phenotypic distributions to have zero mean and unit variance, and focus purely on shape. This operation neutralizes the scale and background artifacts inherent in the dye-labeling and imaging approaches central to many cell biological assays. But there is a danger that normalization might cause initially distinct distributions to collapse onto a single curve, thus throwing the baby out with the bathwater. Our key finding is that, for a broad range of phenotypes, normalized distributions do not collapse; instead, perturbations cause robust changes to their shapes. By detecting these changes, we can screen for candidate genes involved in a variety of cellular processes.

As a practical matter, the fact that we have been able to exploit cell-to-cell variability in this way without needing to understand its mechanistic basis implies that our methods are broadly applicable. However, our results also raise questions of a fundamental nature. What we observe as the individuality of cells in their response to perturbations might be intrinsically stochastic; but it might also reflect a prior heterogeneity of biochemical and biophysical cell states in the population, arising from autonomous factors such as transcriptional, metabolic, and cytoskeletal variations, or higher-order effects involving cell-to-cell communication and coordination. Disentangling these possibilities is an important problem in its own right: the more we discover about the origins of cell-to-cell variability, the better will we understand the secret lives of single cells.

## Methods

### A Z-score to quantify shape changes of phenotypic distributions

We start with a list of values of some phenotype of interest measured in any well. For each data point, we subtract the mean and divide by the standard deviation, resulting in a distribution with zero mean and unit variance. We then compare the normalized distributions from two wells by using the Kolmogorov-Smirnov (KS) test statistic *D*: the maximum vertical distribution between the cumulative distribution functions [32], [33]. If the two sampes have *N*_1_ and *N*_2_ *­* data points, the effective sample size is given by 1/*N_e_*  =  1/*N*_1_ + 1/*N*_2_, and the final statistic is:



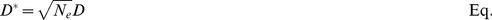
(Eq.\; 1)The Z-score itself is calculated by quantifying the shape deviation from negative controls. For each negative control well, we calculate its D^*^ value against all 300 wells of a slide; this 300-length vector is normalized to have zero mean and unit variance. Arranging each such vector as a row of a 30×300 matrix, the average of each column represents the shape deviation of the corresponding well. In practice we first removed the 10 worst-scoring negatives so that we could use them to estimate false-positive rates. This left a 20×300 matrix shown as a heat map in Figure 3A, with lighter boxes indicating higher shape deviations. The Z-score of any well is the average value of the corresponding column; the higher the score, the greater the shape deviation.

### Assessing statistical power from triplicate data

Each gene is tested in triplicate, and therefore assigned three Z-scores. At a given Z-score threshold, genes can be classified into four bins: occurring zero, one, two, or three times above that threshold. Each bin contains some to-be-determined combination of negatives and true hits. We assume a fraction *h* of all tested genes to be true hits. At a fixed threshold, we assume hits to have a Gaussian distribution of false-negative (FN) rates with mean 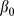 and variance 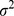, while negatives have a false-positive (FP) rate 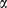. Under these assumptions, genes fall into triplicate bins as follows:



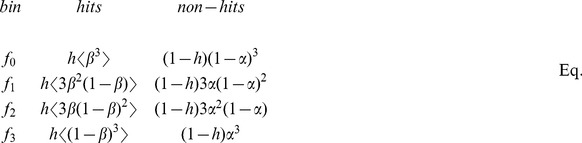
(Eq.\;2)


where the brackets 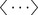 denote averages over the distribution of 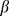 values, easily computed under the Gaussian assumption. We next estimate the values of 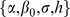 which best describe the observed triplicate bin data. At each threshold, four normalized bins correspond to three degrees of freedom, while we are trying to estimate four unknown parameters. However, since *h* is a constant independent of threshold, the system is “almost” determined, and we are able to find a unique solution with a non-zero least-squares error. We can thus find 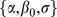 as a function of the threshold, as well as a fixed value of *h*, for each parameter (Fig. S3 in File S1).

## Supporting Information

File S1 Contains the following tables and figures: **Table S1.** Feature descriptions. **Figure S1.** Slide layout and positional artifacts. **Figure S2.** Presence of reproducible hits. **Figure S3.** Performance inferred from triplicate data. **Figure S4.** Cell states and cell-to-cell variability.
(PDF)

## References

[1] Levsky JM, Singer RH (2003) Gene expression and the myth of the single cell. Trends Cell Biol 13: 4. 12480334 10.1016/s0962-8924(02)00002-8

[2] Raser JM, O'Shea EK (2005) Noise in gene expression: origins, consequences, and control. Science 309: 2010–2013. 16179466 10.1126/science.1105891 PMC1360161

[3] Balaban NQ, Merrin J, Chait R, Kowalik L, Leibler S (2004) Bacterial Persistence as a Phenotypic Switch. Science 305: 1622–1625. 15308767 10.1126/science.1099390

[4] Chang HH, Hemberg M, Barahona M, Ingber DE, Huang S (2008) Transcriptome-wide noise controls lineage choice in mammalian progenitor cells. Nature 453: 544–547. 18497826 10.1038/nature06965 PMC5546414

[5] Bahar R, Hartmann CH, Rodriguez KA, Denny AD, Busuttil RA, et al (2006) Increased cell-to-cell variation in gene expression in ageing mouse heart. Nature 441: 1011–1014. 16791200 10.1038/nature04844

[6] Slack MD, Martinez ED, Wu LF, Altschuler SJ (2008) Characterizing heterogeneous cellular responses to perturbations. Proc Natl Acad Sci USA 105: 19306–19311. 19052231 10.1073/pnas.0807038105 PMC2614757

[7] Snijder B, Sacher R, Rämö P, Damm EM, Liberali P, et al (2009) Population context determines cell-to-cell variability in endocytosis and virus infection. Nature 461: 520–523. 19710653 10.1038/nature08282

[8] Ozbudak EM, Thattai M, Kurtser I, Grossman AD, van Oudenaarden A (2002) Regulation of noise in the expression of a single gene. Nat Genet 31: 69–73. 11967532 10.1038/ng869

[9] Elowitz MB, Levine AJ, Siggia ED, Swain PS (2002) Stochastic gene expression in a single cell. Science 297: 1183–1186. 12183631 10.1126/science.1070919

[10] Raser JM, O'Shea EK (2004) Control of stochasticity in eukaryotic gene expression. Science 304: 1811–1814. 15166317 10.1126/science.1098641 PMC1410811

[11] Bakal C, Aach J, Church G, Perrimon N (2007) Quantitative morphological signatures define local signaling networks regulating cell morphology. Science 316: 1753–1756. 17588932 10.1126/science.1140324

[12] Keren K, Pincus Z, Allen GM, Barnhart EL, Marriott G, et al (2008) Mechanism of shape determination in motile cells. Nature 453: 475–480. 18497816 10.1038/nature06952 PMC2877812

[13] Geva-Zatorsky N, Rosenfeld N, Itzkovitz S, Milo R, Sigal A, et al (2006) Oscillations and variability in the p53 system. Mol Syst Biol 2: 2006.0033. 10.1038/msb4100068 PMC1681500 16773083

[14] Gupta GD, Dey D, Swetha MG, Ramalingam B, Khader S, et al.. (2014) Cell heterogeneity reveals a hierarchy of molecular players underlying parallel endocytic pathways. In revision, PLoS ONE, Manuscript ID PONE-D-13-36960R1.

[15] Lum L, Yao S, Mozer B, Rovescalli A, Von Kessler D, et al (2003) Identification of Hedgehog pathway components by RNAi in Drosophila cultured cells. Science 299: 2039–2045. 12663920 10.1126/science.1081403

[16] Foley E, O'Farrell PH (2004) Functional dissection of an innate immune response by a genome-wide RNAi screen. PLoS Biol 2: E203. 15221030 10.1371/journal.pbio.0020203 PMC434151

[17] Hamilton B, Dong Y, Shindo M, Liu W, Odell I, et al (2005) A systematic RNAi screen for longevity genes in C. elegans. Genes Dev 19: 1544–1555. 15998808 10.1101/gad.1308205 PMC1172061

[18] Zhou H, Xu M, Huang Q, Gates AT, Zhang XD, et al. (2008) Genome-scale RNAi screen for host factors required for HIV replication. Cell Host Microbe 4, : 495–504. 10.1016/j.chom.2008.10.004 18976975

[19] Kiger AA, Baum B, Jones S, Jones MR, Coulson A, et al (2003) A functional genomic analysis of cell morphology using RNA interference. J Biol 2: 27. 14527345 10.1186/1475-4924-2-27 PMC333409

[20] Agaisse H, Burrack LS, Philips JA, Rubin EJ, Perrimon N, et al (2005) Genome-wide RNAi screen for host factors required for intracellular bacterial infection. Science 309: 1248–1251. 16020693 10.1126/science.1116008

[21] Goshima G, Wollman R, Goodwin SS, Zhang N, Scholey JM, et al (2007) Genes required for mitotic spindle assembly in Drosophila S2 cells. Science 316: 417–421. 17412918 10.1126/science.1141314 PMC2837481

[22] Guo Y, Walther TC, Rao M, Stuurman N, Goshima G, et al (2008) Functional genomic screen reveals genes involved in lipid-droplet formation and utilization. Nature 453: 657–661. 18408709 10.1038/nature06928 PMC2734507

[23] Neumann B, Walter T, Hériché JK, Bulkescher J, Erfle H, et al (2010) Phenotypic profiling of the human genome by time-lapse microscopy reveals cell division genes. Nature 464: 721–727. 20360735 10.1038/nature08869 PMC3108885

[24] Collinet C, Stöter M, Bradshaw CR, Samusik N, Rink JC, et al (2010) Systems survey of endocytosis by multiparametric image analysis. Nature 464: 243–249. 20190736 10.1038/nature08779

[25] Brideau C, Gunter B, Pikounis B, Liaw A (2003) Improved statistical methods for hit selection in high-throughput screening. J Biomol Screen 8: 634–647. 14711389 10.1177/1087057103258285

[26] Malo N, Hanley JA, Cerquozzi S, Pelletier J, Nadon R (2006) Statistical practice in high-throughput screening data analysis. Nat Biotechnol 24: 167–175. 16465162 10.1038/nbt1186

[27] Mayor S, Pagano RE (2007) Pathways of clathrin-independent endocytosis. Nat Rev Mol Cell Biol 8: 603–612. 17609668 10.1038/nrm2216 PMC7617177

[28] Gupta GD, Swetha MG, Kumari S, Lakshminarayan R, Dey G, et al (2009) Analysis of endocytic pathways in Drosophila cells reveals a conserved role for GBF1 in internalization via GEECs. PLoS One 4: e6768. 19707569 10.1371/journal.pone.0006768 PMC2728541

[29] Howes MT, Mayor S, Parton RG (2010) Molecules, mechanisms, and cellular roles of clathrin-independent endocytosis. Curr Opin Cell Biol 22: 519–527. 20439156 10.1016/j.ceb.2010.04.001

[30] Traub LM (2009) Tickets to ride: selecting cargo for clathrin-regulated internalization. Nat Rev Mol Cell Biol 10: 583–596. 19696796 10.1038/nrm2751

[31] Guha A, Sriram V, Krishnan KS, Mayor S (2003) Shibire mutations reveal distinct dynamin-independent and -dependent endocytic pathways in primary cultures of Drosophila hemocytes. J Cell Sci 116: 3373–3386. 12857788 10.1242/jcs.00637

[32] Press WH, Teukolsky SA, Vetterling WT, Flannery BP (1992) Kolmogorov-Smirnov Test. In: Numerical Recipes in C*, * *ed.* Camridge: Cambridge University Press. pp. 623–628.

[33] Massey F (1951) The Kolmogorov-Smirnov test of goodness of fit. J Am Stat Assoc 46: 68.

[34] Ashburner M, Ball CA, Blake JA, Botstein D, Butler H, et al (2000) Gene ontology: tool for the unification of biology. Nat Genet 25: 25–29. 10802651 10.1038/75556 PMC3037419

[35] Sacher R, Stergiou L, Pelkmans L (2008) Lessons from genetics: interpreting complex phenotypes in RNAi screens. Curr Opin Cell Biol 20: 483. 18602470 10.1016/j.ceb.2008.06.002

