# Supplementary material for: Exploiting Cell-To-Cell Variability To Detect Cellular Perturbations
Source: PLoS One. 2014 Mar 4;9(3):e90540. doi: 10.1371/journal.pone.0090540 (PMC3942435; doi:10.1371/journal.pone.0090540)
Supplement: File S1 — Contains the following tables and figures: Table S1. Feature descriptions. Figure S1. Slide layout and positional artifacts. Figure S2. Presence of reproducible hits. Figure S3. Performance inferred from triplicate data. Figure S4. Cell states and cell-to-cell variability. (PDF) [file pone.0090540.s001.pdf]

Supporting Information for:

Exploiting cell-to-cell variability to detect cellular perturbations

Gautam Dey, Gagan D. Gupta, Balaji Ramalingam, Mugdha Sathe, Satyajit Mayor, Mukund Thattai

Table S1: Feature descriptions
Figure S1: Slide layout and positional artifacts
Figure S2: Presence of reproducible hits
Figure S3: Performance inferred from triplicate data
Figure S4: Cell states and cell-to-cell variability

Table S1: Feature descriptions

Feature	Description	Biological relevance
Fint1 (I1)<br>Tint1 (I5)	Average intensity	Total endocytosed cargo, including out-of-focus structures
Fint2 (I2)<br>Tint2 (I6)	Average intensity after coarse-grained local background subtraction	Intensity of endosomes, excluding out-of-focus structures
Fint3 (I3)<br>Tint3 (I7)	Average intensity after fine-grained local background subtraction	Intensity of bright, in-focus endosomes
Fint4 (I4)<br>Tint4 (I8)	Fraction of cell with non-zero intensity after fine-grained local background subtraction	Density of endocytosed material
Rto1–3 (I9–I11)	Tint1,Tint2,Tin3, normalized to surface levels of Tfr as labeled by Okt9	Ratio of internalized to surface levels of Tfr
Okt (I12)	Average intensity of Okt9	Surface levels of Tfr
Fmph1 (G1)<br>Tmph1 (G6)	Average size of discrete objects within the cell after thresholding	Endosome size
Fmph2 (G2)<br>Tmph2 (G7)	Area occupied by discrete objects as a fraction of cell size	Density of endocytosed material; similar to Fint4 and Tint4
Fmph3 (G3)<br>Tmph3 (G8)	Average circularity of discrete objects	Simple descriptor of endosome morphology
Fnum (G4)<br>Tnum (G9)	Total number of discrete objects	Number of endosomes
Fclc (G5)<br>Tclc (G10)	Fraction of discrete FDex (Tfr) objects that colocalize with Tfr (FDex) objects	Fraction of FDex (Tfr) endosomes that contain Tfr (FDex)
CellSize (G11)	Area of a cell as marked by surface Okt9	Estimate of cell size
NucSize (G12)	Area of nucleus labeled by DAPI	Estimate of nuclear size
NucCirc (G13)	Circularity of the nucleus	Simple descriptor of nuclear morphology
NucFluct (G14)	Standard deviation of the distance from the centroid of the nucleus to its perimeter	Ruffling of nuclear envelope
NucDist (G15)	Distance of nuclear centroid from cell centroid	Localization of nucleus

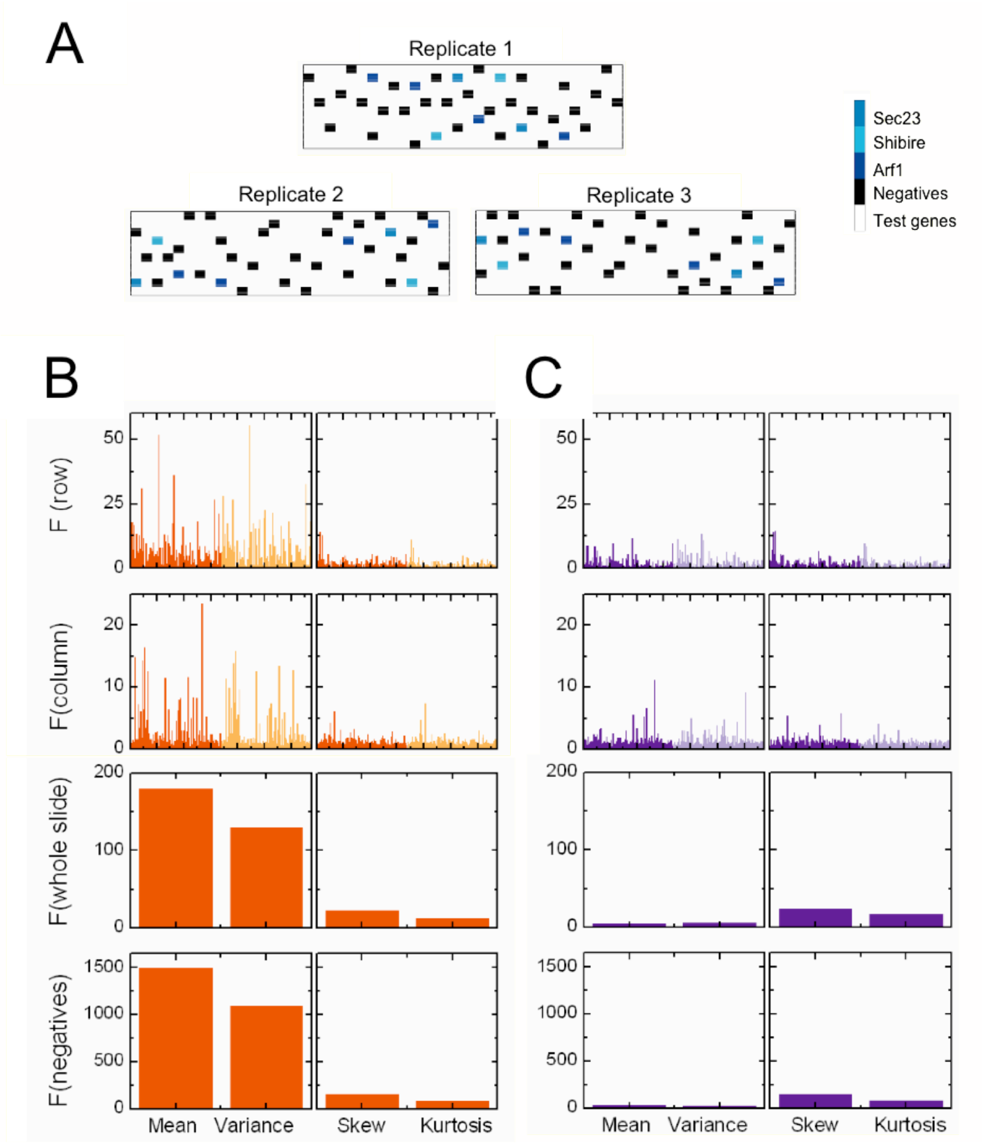

Figure S1. Slide layout and positional artifacts. **(A)** Triplicate layout of negative (black) and positive (blue) control wells on the 10x30 slide. Test genes (white) were assayed in triplicate, with positions scrambled to minimize the confounding effects of positional artifacts. **(B,C)** ANOVA F-statistic for four descriptors (mean, variance, skewness and kurtosis) of the population distributions of (B) intensity feature I3 and (C) geometry feature G3. The top two panels represent the F-statistic for inter-row or inter-column variance compared to within-row or within-column variance on a single slide. Each bar is a single slide, with 84 slides in total. Within row data are summarized in Fig. 2E. The third panel from the top represents the F-statistic for inter-slide variance compared to within-slide variance, using all wells on each slide. The bottom panel shows the result when this is restricted to negatives alone.

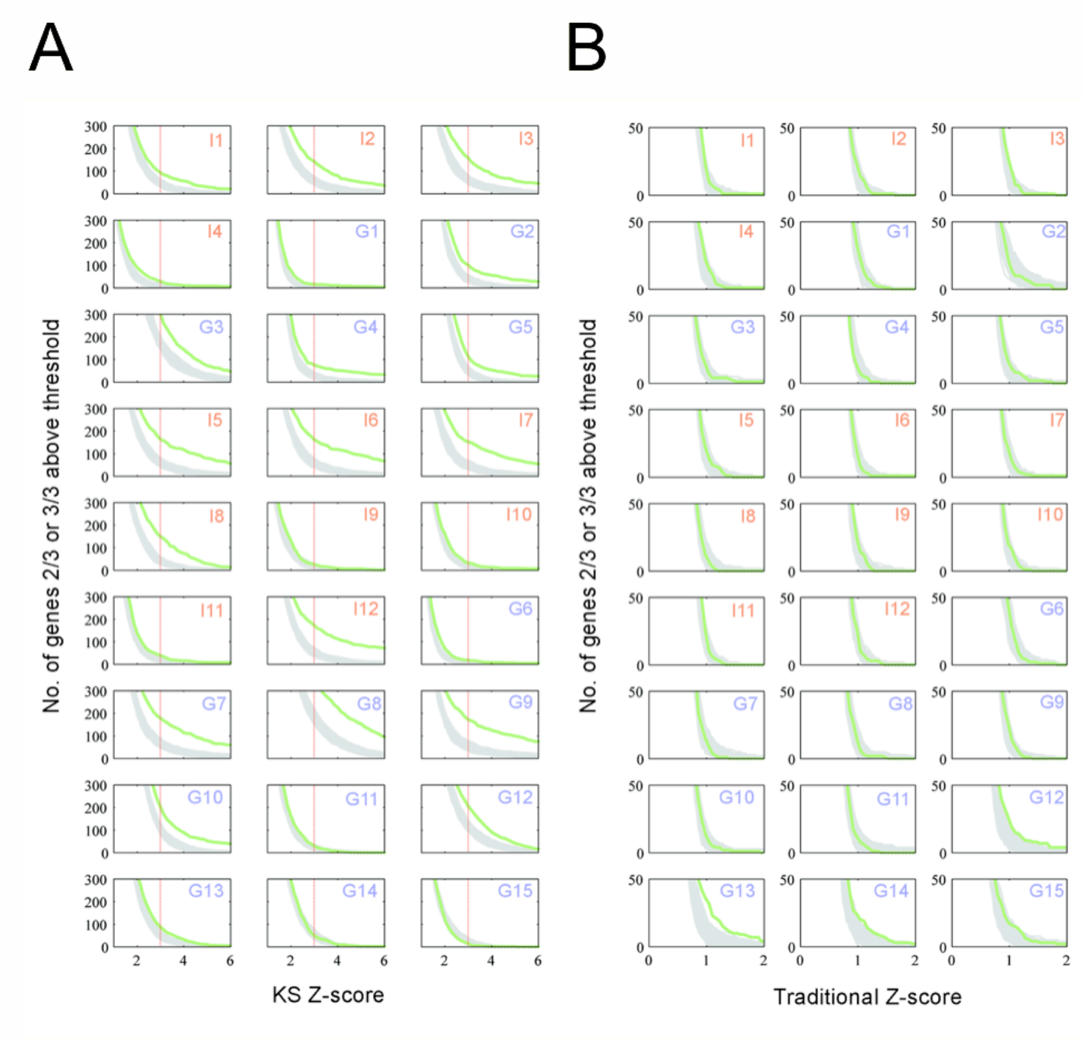

Figure S2. Presence of reproducible hits. The number of genes that occur two or more times above each Z-score threshold, for all 27 features. The green curve shows the number of genes selected from the screen; the grey band represents the upper and lower limits of number of genes selected from 1000 randomly permuted datasets. **(A)** Results using the KS-based Z-score; we used a Z-score cutoff of 3 (red line) to select hits. **(B)** Results using the 'traditional' Z-score. For almost all features, the number of genes selected using the 'traditional' Z-score lies completely within the grey band expected by chance.

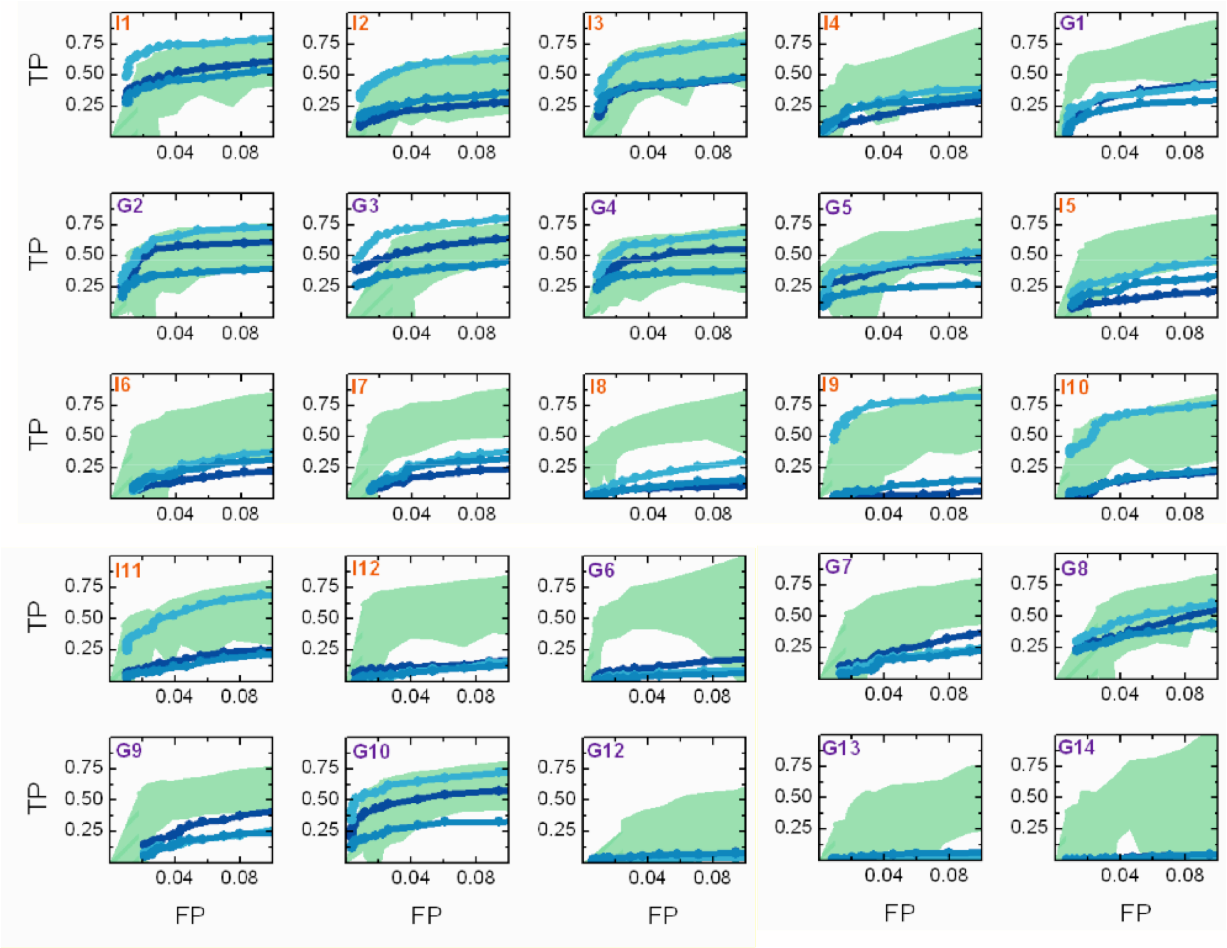

Figure S3. Performance inferred from triplicate data. For each feature we calculate the values {α, β_0, σ, h} which best fit the observed triplicate data as the Z-score threshold is varied (Materials and Methods: Assessing statistical power from triplicate data). Intensity features are labeled orange, geometric features are labeled purple. (The inference procedure fails to converge to a solution for features G11 and G15; results for the remaining 25 features are shown.) As the Z-score threshold is decreased (made less stringent) the true positive (TP = 1–β_0) and false positive (FP = α) rates both increase. The green band shows the TP range (1–$\beta_0 \pm \sigma$) as a function of FP (α). The TP and FP rates can be directly observed for the set of positive control genes; these are plotted as blue lines (light: Shibire, medium: Arf1, dark: Sec23). Note that each of these control genes is only a positive control for some subset of features. In such cases the observed TP vs. FP curve matches well with the inferred band (e.g. for feature I1); in other cases these genes can be thought of as negative controls, and the observed curve will be close to the line TP = FP (e.g. for feature I12).

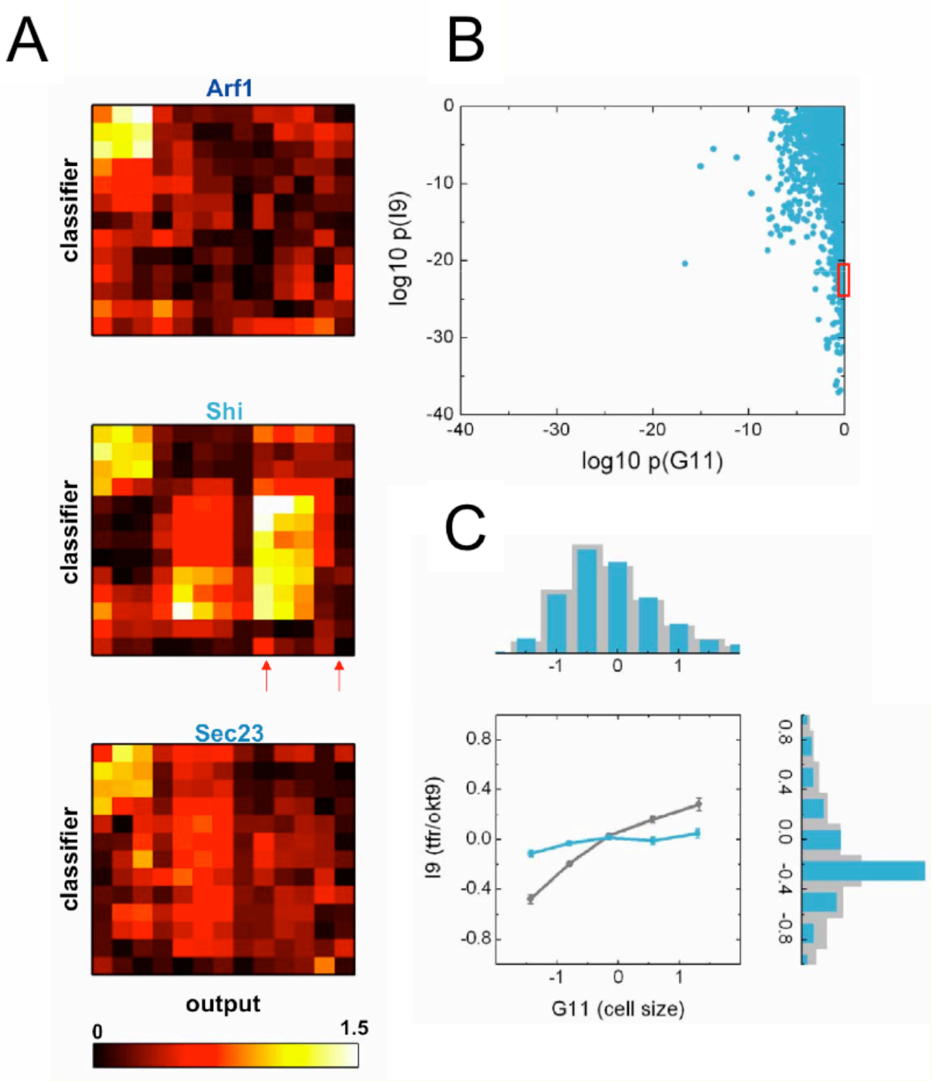

Figure S4. Cell states and cell-to-cell variability. **(A)** A 13x13 heatmap of the correlation score Δy_{ij}, for three sets of positive control genes (Arf1, Shibire, and Sec23). The Shibire panel shows two features selected as a possible classifier-output pair: the right arrow shows G11 (cell size), which as a low on-diagonal score; the left arrow shows I9, which has a high score when G11 is used as the classifier. **(B)** Scatterplot of all pairwise KS-test p-values for all negative control wells against all Shibire wells, for feature I9 (x-axis) and G11 (y-axis); I9 is strongly perturbed but G11 is not. **(C)** We pooled data for cells from wells within the red box in (B). Histograms show the distribution of G11 (top) and I9 (right) for cells from negative control wells (grey) and Shibire-RNAi wells (blue). As expected for wells within the red box, G11 distributions do not change upon perturbation, but I9 distributions do. We place cells into five bins based on their classifier (G11) values, and calculate the mean output (I9) in each bin. This classifier-output curve is different between negatives and Shibire-RNAi-treated cells. That is, the response to RNAi is at least partly dependent on cell size.
